# Non‐redox Lewis Acids Supported Pd‐Catalyzed C—C /C—H Activation and Accession to Produce Pharmaceutical Intermediates

**DOI:** 10.1002/open.202500399

**Published:** 2026-03-26

**Authors:** Ahmed M. Senan, Senem Akkoc, A. M. Abdulkarem, Sadeq K. Alhag

**Affiliations:** ^1^ Department of Pharmacy Faculty of medicine and health Science Mahweet University Al‐Mahweet Yemen; ^2^ Department of Chemistry Faculty of Science Taiz University Taiz Yemen; ^3^ Department of Basic Pharmaceutical Sciences Faculty of Pharmacy Suleyman Demirel University Isparta Türkiye; ^4^ Faculty of Engineering and Natural Sciences Bahçeşehir University Istanbul Türkiye; ^5^ Department of Physics College of Education and Applied Sciences Sana’a University Sanaa Yemen; ^6^ Department of Biology College of Science and Arts King Khalid University Muhayl Asser Saudi Arabia

**Keywords:** C—H activation, catalysis, Lewis acid, oxidation, synthesis

## Abstract

Palladium is a simple and versatile redox‐active metal that plays a crucial role in the oxidation reactions for organic synthesis and production of pharmaceutical intermediates. Notably, there has been significant progress with Lewis acids (LAs), which are non‐redox metal ions promoting Pd(II)‐catalyzed oxidation via C—H and C—C bond activation. A new catalytic species is formed through the strong interaction between a Lewis acid and palladium as dimer catalyst. This active complex in this system, which is a Lewis acid promoter, facilitates a Wacker‐type oxidation reaction via the activation of C—H and/or C—C bonds. This system of dimer catalysis is based on the key active species for oxidation reactions in pharmaceutical production. The Pd(II)/LA‐catalytic oxidation of olefins and N‐heteroaromatic alkylation we developed offers a robust method for generating intermediate compounds for drug discovery. This study demonstrates that non‐redox Lewis acid‐promoted Pd catalysis is highly effective in synthesizing chemically diverse compounds featuring hydrogen‐bonding acceptors and donors (HBA/HBD). These functional groups are crucial for interactions with biological targets, making the resulting molecules strong candidates for development as active pharmaceutical ingredients. Their potential applications span pharmaceutical sciences, medicinal delivery systems, and cosmetic formulations.

## Graphical abstract

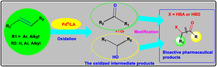

## 1 Introduction

Redox metal ions are used in functionalizing C—H and C—C bonds in organic molecules, which offer highly promising protocols to synthesize versatile molecules, functional materials, and pharmaceuticals. Palladium is more attractive than the other transition metal ions modified for C—H and/or C—C activation [1]. Many palladium complexes are based on redox metal ions in synthetic methodologies through the redox reactions by an organic acid or Lewis acids. However, re‐oxidizing the Pd(0) occurs with the oxidants or promoters, which gives the best selectivity of the molecule's activation [2]. Therefore, several catalytic methods are employed in the synthesis of biologically active molecules such as imidazole derivatives and/or indolyl‐containing N‐hetero‐compounds. Thus, a reaction undergoes oxidative coupling procedures of multi‐step production of an active pharmaceutical ingredient. The N‐hetero (Bi)‐aryl structure has attracted much attention for the merging of two hetero‐aromatics, making it one of the most important areas of research on carbon−carbon bond‐forming new structures of pharmaceuticals, ligands, and organic synthetic intermediates involving cosmetics and medicinal delivery [3, 4, 5, 6]. Lucas Montero has reported that the photo‐peroxidation conversion of high oleic sunflower oils to an *α*,*β*‐unsaturated ketone‐containing triglyceride via a two‐step, one‐pot process. Schenck‐Ene reported in his published article on the oxyfunctionalization of unsaturated triglyceride at the allylic position, then it's used to synthesize and obtain (N—C=O)‐polymers [7, 8, 9, 10, 11]. Recently, Grubbs [12], Sigman [13], and Kaneda [14] have reported the Wacker‐type procedure of internal olefin to synthesize ketones by developing a system for oxidation and often using Pd(II) catalysis of internal olefin oxidation with LA co‐catalysis. Remarkably, the procedure required convenient conditions such as atmospheric pressure of oxygen and room temperature [15, 16, 17, 18].

Various catalytic methods have been explored in organic chemistry, and transition metal catalysis stands out together with proper catalytic strategies in the pharmaceutical industry. Further, present Lewis acid/Pd^2+^ shows more versatile reactivity and higher catalytic efficiency in most chemical processes of bioactive products. The C—H/C—C bond activation is faced with a greater challenge than the other bonds, such as C—O and C—X (Cl, Br, I) and C—N bonds, due to the higher energy and immersive spread with often marginal chemical differences in organic compounds. For C—H or C—C activation, there are two early‐stage different kinds of reactions, depending on the intermolecular reaction of substrates and the interaction between substrates and the catalytic reagent, thus ultimately promoting reactivity and selectivity [19]. This study focused on Lewis acid, a mixture with palladium as a dimeric heterobimetallic metal catalyst, which facilitates inert bond activation and product‐complexity construction. Adding Lewis acid to palladium proves the oxidation successful and increases the progress of achieving C—H and C—C bond activation during the last decade. Editing the mechanisms of redox reactions by the enzyme catalysts, a very important characteristic for oxidation, has not been fully understood yet, which is why there are difficulties in controlling the active pharmaceutical intermediates in diverse enzyme catalysts. The development of catalytic methods by using non‐redox metal ions (Lewis acids) with transition metals, which has shown very important strategies, including Pd‐(II) catalysts, is the improvement of the catalytic efficiency of an active pharmaceutical ingredient and stoichiometric oxidations with active redox metal ions [18, 20, 21, 22].

## 2 The Oxidation‐Addition Cyclization via C—H/C—C Activation by Pd(II)/Cu(II) Catalyst

**FIGURE 1:**
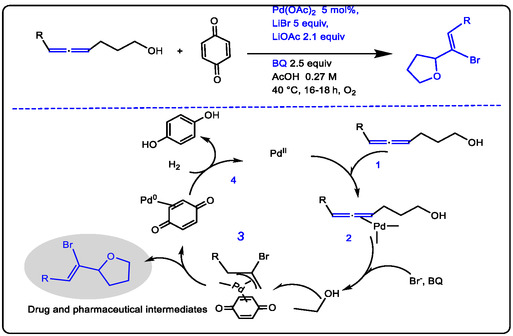
Intermolecular Pd(II) catalyzed 1,2‐addition cyclization via re‐oxidizing Pd(0) to the active Pd(II) species to produce the tetrahydrofuran, its modified form reference [23] and Licensed Number 96213840477716 provided from the American Chemical Society.

Palladium catalyst has been applied in oxidative/isomerization and improving the catalytic C—H/C—C activation with Cu(II) as an additive. In this case, Cu(II) acts as a stoichiometric co‐catalyst and oxidant to re‐oxidize Pd(0) to the active Pd(II) species. This is analogous to its role in dehydrogenative processes like catalyzed 1,2‐additions and the Heck reaction. Separately, the (*π*‐allene)palladium complex is reactivated to form a (*π*‐allyl)palladium intermediate (step 3 in Figure 1) [23, 24, 25].

**FIGURE 2:**
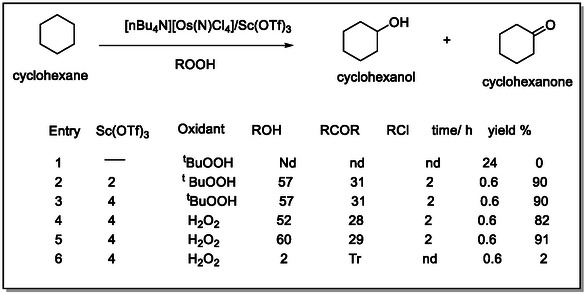
Catalytic oxidation of cyclohexane by [*n*Bu_4_N][Os(N)Cl_4_]/Sc(OTf)_3_/ROOH; (1) using the [*t*Bu_4_ N]Os(N)Cl_4_] catalyst 1.25 × 1000 M in absence Scandium, then, (2) optimized condition; Sc(OTf)_3_ added 5.0 x 10^−3^, in the replaced of [*t*Bu_4_ N]Os(N)Cl_4_] by H_2_O_2_ the entries of 4–6, and change the oxidant ligands. In entry 6 was added H_2_O = to mixture reaction in absence of [*t*Bu_4_ N]Os(N)Cl_4_ [30, 31].

Halides attack alkenes to give (*π*‐allyl) palladium complexes. Palladium is present as a σ‐allyl‐palladium intermediate and coordinated in situ with p‐benzoquinone to form the *π*‐allyl complex as potential pharmaceuticals, which produce tetrahydrofuran and an active pharmaceutical ingredient (API), actually drugs, such as in (Figure 1) [26, 27, 28]. For the achievement of an efficient catalytic cycle of the Pd(II) acetate, some of the organic substances were selected to prohibit the formation of palladium black; these ligands decrease the oxidizing power of the Pd(II) species. For example, using trifluoroacetic acid (TFA) as a solvent and/or adding copper (II) as an additive matter to the Pd(OAc)_2_ catalyst for benzene hydroxylation to obtain biphenyl and phenol as antioxidants. Furthermore, the increase in catalytic efficiency was by adding 2,2‐pyridine or phenanthroline as the ligand, which makes Pd(OAc)_2_ inactive without Pd(II) hydride in benzene hydroxylation [29, 30]. Numerous publications reported the detection of the activation of intermediate redox metals like Os(N)Cl_4_ with Lewis acids such as Sc(OTf)_3_, Zn^2+^, and Al^3+^, and, for example, adding 2 mM of Sc(OTf)_3_ to 1 mM of Pd^2+^ significantly increased the catalytic activity of Pd(OAc)_2_ in producing fine chemicals, which was determined by UV/VIS and GC spectroscopy. The active intermediate absorbed by the catalytic oxidation of cyclohexane is good application in medicinal fields, and UV‐spectral changes were observed in the absence of Sc(OTf)_3_, but the formation of the intermediate complex [Os(N)Cl_4_]^‐^ with ^t^BuOOH. The achieved processing of catalytic oxidation of cyclohexane was determined by GC/GC–MS spectrometer. Lewis acid‐like Sc(OTf)_3_ showed the highly activated key to both producing pharmaceutical intermediate steps (see Figure 2) [31].

**FIGURE 3:**
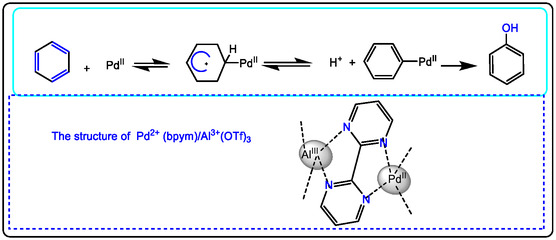
Pd^II^(bpym)/Al(OTf)_3_‐catalyzed benzene hydroxylation to phenol as antioxidant and antiseptic, Adapted from reference [30]. Copyright provided by John Wiley and Sons, Chemistry ‐ An Asian Journal, license Number 6212670104770.

This study scans the development of non‐redox metal ions (Lewis acids) in the oxidation of cyclic compounds in chemical procedures of pharmaceuticals via C—H activation with Pd (II)‐catalyst; Lewis acid improves the catalytic efficiency of stoichiometric oxidation with active/inactive redox metal ions, even in high oxidation states of drug discovery. The active metal, like oxo, hydroxo, and hydroperoxide, is observed in the intermediates of oxidation, and the increase in positive net charge is made by activating metal by electron transfer and protonation (Brønsted acid). Producing some of the encouraged pharmaceutical products, we explored that with the Brønsted acid/Lewis acid as co‐catalysts, metal ions promoted catalytic oxidation with transition metals (redox metal ions) like Pd(II). In particular, this information is used to design a new catalyst system for fine chemicals oxidizing and exploring the efficiency of Lewis acids such as trivalent (Al^3+^, Sc^3+^), bivalent (Mg^2+^, Zn^2+^), and/or monovalent (Na^+^, Li^+^) with a palladium catalyst. For example, Lewis acid added to Pd(bpym)_2_ catalyzed hydroxylation of benzene into pharmaceutical candidates, where Pd^2+^ alone is inactive (Figure 3) [18, 22, 31, 32, 33, 34, 35, 36, 37].

To C—H bond activation on benzene, which could be significantly enhanced by Pd(II) species, for improving chemical processes such as in hydroxylation of alkenes or phenolytion of benzene for producing effective microorganisms’ agents. The result of this reactivity was caused by the binding of Lewis acid to two remote nitrogen atoms of the (bpym)_2_ ligand, the structure of the intermediate complex proposed for Pd(II)/Al(III) (see in Figure 3).

Thus, recovering the ability of C—H activation and improving the redox potential of the Pd(II) species depended on increasing the net charge of the Al(III)‐bpym‐Pd(II) unit as an intermediate complex in the catalytic system. Most recently, it was further found that Sc(III) as a nonredox metal ion can promote Wacker‐type oxidation of olefins and vegetable oils completely and very well, even better than Cu(II), and using UV–vis spectroscopy for comparing Sc(III) with Cu(II), both of which are employed for accelerating Pd(II)‐catalyzed olefin isomerization and/or oxidation, so that the redox properties of Cu(II) are not necessary [35, 36]. These findings clearly show that the acidity and Lewis properties of Cu (II) may play a significant role in versatile Pd(II)‐catalyzed C—H bond activations. It's employed as a co‐catalyst or stoichiometric oxidant to re‐oxidize the reduced Pd(0) [37].

This review has covered several novel strategies for using redox metal ion‐catalyzed C—H bond activation in pharmaceuticals and bioactive molecule synthesis. For example, Lewis acids (non‐redox metal ions) have accelerated Pd(II)‐catalyzed oxidative addition of the aryl bromide and the other cross‐coupling reactions of indoles with olefins and/or acrylates through dioxygen activation. Unexpectedly, the synthesis of 3,3‐bis(indolyl)propanoic esters as one category of 3,3‐bis(indolyl)methane derivatives in the occurrence of a Sc(III)/Pd(II) catalyst. Using Pd(OAc)_2_ alone as the catalyst only delivered olefination products [37]. Redox transition metal ions like Pd(II) can promote the synthesis of bis(indolyl)methane molecules as good drugs applicable for antiviral activity, antibacterial activity, anti‐inflammatory activity, and anticarcinogenic properties. Generally, these types of molecules have been synthesized by the condensation reaction known as the chalcone reaction between aldehydes and/or ketones with N‐heterocyclic carbons and/or in the addition of indoles to the alkenes or to alkynes, which supported the result of the reaction [35, 36, 37, 38]. Lewis acids are increasing the reactivity of non‐heme redox catalysts toward the epoxidation of (double bonds) olefins. In the epoxidation reaction of olefins, the reduction of Pd (II) intermediates to Pd (0) is a key elementary step. The Pd(II) as a redox metal catalyst engaged with PhCH_2_C(CH_3_)_2_OOH (MPPH). The Lewis acid property of this additive oxidant ligand is used as a co‐catalyst. The mechanism was proposed for different unsaturated hydrocarbon oxidation in the presence of MPPH to differentiate metal‐based free radicals of chemical intermediates. When the Pd(II) redox‐metal ions replaced the Mn(II) catalyst in the oxidation process of olefins, it enhanced the catalytic sensitivity in providing good oxidation conditions for olefins in mixed solvents [37, 38, 39, 40].

## 3 Wacker‐Type Oxidation of Olefins in Pharmaceutical Synthesis

### 3.1 Pd(II)/Cu(II)‐Wacker Type Oxidation Reaction of Olefins

**FIGURE 4:**
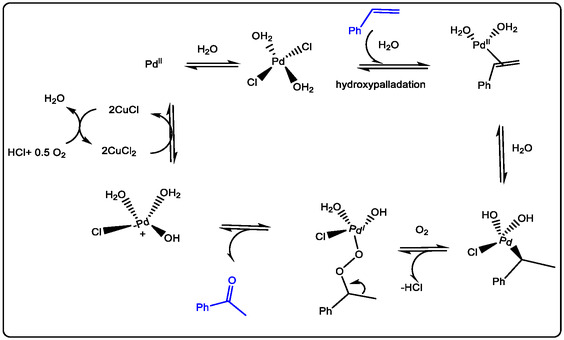
Mechanism of Wacker–Tsuji oxidation of olefins by Pd^II^/Cu^II^ catalyst, modified from references [36] Order License ID: 1702154‐1, is provided by the ROYAL SOCIETY OF CHEMISTRY.

The classical Pd(II)/Cu(II)‐catalyzed Wacker oxidation of olefins provides a practical route for producing valuable keto‐chemical intermediates and remains a vital tool in synthetic chemistry. In this process, a copper co‐catalyst facilitates the re‐oxidation of Pd(0) under aerobic conditions by utilizing oxygen as the terminal oxidant. These processes convert the olefins to their oxo‐products, enabling a catalytic cycle known as the Wacker–Tsuji oxidation. This transformation offers a practical route to valuable carbonyl intermediates, which are essential building blocks in the pharmaceutical industry, cosmetics, and drug delivery systems (see Figure 4) [36].

The mechanism of Pd(II) catalysis applied in Wacker‐type oxidation with redox‐metal ions such as Cu^2+^ has also been extensively investigated with Lewis acids as non‐redox metals, which could improve the oxidation of olefin. In this phenomenon, numerous articles have been published; for example, the non‐redox metal ions,such as Lewis acids like Sc(OTf)_3_, promoted Wacker‐type oxidation for obtaining excellent yields of keto‐alkyls and/or aryls. That was found to be a good potential drug, which has been selected and modified as a candidate in the last few years. In addition, the Cu(II) with stronger Lewis acidity properties can play a significant role, and besides its redox properties, it also gives great support to Pd(II)‐catalyzed C—H activation, for which the acidity of the divalent Lewis acid gives reason to increase the potential efficiency ion of Cu^2+^ in oxidation. Wacker–Tsuji oxidation of olefins by PdII/CuII catalyst [18, 36, 37, 41].

### 3.2 Wacker‐Type Oxidation of Olefins by Pd^II^/Lewis Acid

For Wacker‐type oxidation of olefin in Pd(II)/LA catalyst, which was investigated and informed by GC and UV‐Vis spectroscopy, the generation of a new Pd(II)/Sc(III) heterobimetallic complex was the key active species in acetonitrile solution. Adding the trivalent Sc(III) to the Pd(II) species, which makes it more nucleophilic in a mixture solvent, facilitates the attack of water on the olefinic double bond and leads to efficient olefin oxidation. The formation of a new active intermediate of the Sc(OTf)_3_/Pd(OAc)_2_ catalyst is characterized by NMR, GC, and UV–vis spectrum under optimized conditions [41, 42, 43, 44]. This review examines catalysts based on metal ions with specific Lewis acid strengths, such as Sc(III), which can substantially promote Pd(II)‐catalyzed oxidation of olefins, even outperforming Cu(II). This promotional effect is dependent on the strength of the Lewis acid. The kinetics of these reactions were investigated using gas chromatography. Regarding the Lewis acidity of Cu^2+^, adding Cu(OTf)_2_ to Pd(II) plays a significant role in Wacker‐type oxidation; however, it proved less effective than triflate‐based Lewis acids such as Sc^3+^ and Al^3+^ [36].

**FIGURE 5:**
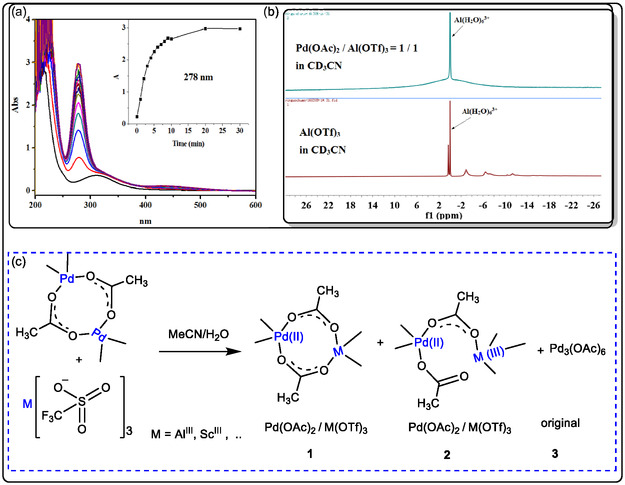
The proposed mechanism of heterobimetallic Al(III) and Pd(II) catalyst: (a) a UV–vis kinetics study of the Pd(II)/Al(III)‐catalyzed conversion of alkenes and (b) ^1^HNMR Spectra of Al(Otf)_3_ alone and Pd(II)/Al(III) together; (c) heterobimetallic M(III) and Pd(II) catalyst in a dimer system, adapted from references [38, 40], (License 96212670902367), Feb 19, 2026, is provided by the American Chemical Society and Copyright Clearance Center.

**FIGURE 6:**
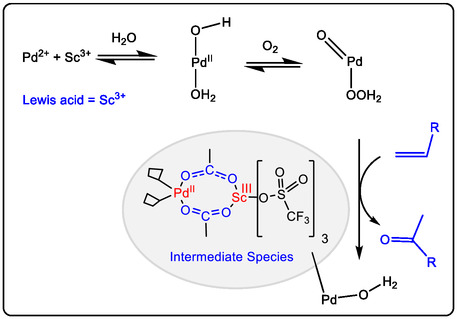
The proposed mechanism of relationship between Lewis acids and the active ox/ hydroxo metal in functionalization pharmaceutical intermediates [14].

In our previous study on Pd(II)‐catalyzed conversion of olefins to their conjugated ketones (or aldehydes). Using UV–vis characterizations of Pd(II)‐catalyzed oxidation of unsaturated hydrocarbons, the Al(OTf)_3_ is added to the acetonitrile solution of Pd(OAc)_2_, which immediately acts to form an intermediate. The result of now complex products is an absorbance maximum at 278 nm (Figure 5a), which has gradually changed to form a stable species with a new band at ∼310 nm. Using UV–vis study for kinetics of adding 1‐octene to a Pd(OAc)_2_/Al(OTf)_3_ mixture in acetonitrile, and having different gradual bands at 278 nm (Figure 5a). The UV–vis spectra studied is identical to the ^1^H NMR spectra analysis of Pd(OAc)_2_ alone and/or Pd^II^/Al(III) catalyst in acetonitrile‐d_3_ (Figure 5b). The water is added in a small ratio of cosolvent with acetonitrile. Lewis acid as non‐redox ion metal cocatalyst to regenerate the new Pd(0)‐catalyst with molecular oxygen; the key step of nucleophilic attack by water (Markovnikov addition) and the olefin coordination to Pd(II). The next step showed the β‐hydride elimination and Pd(0) reoxidation by Sc(III) or Al(III) and regenerating Pd(II) with O_2_ again [37, 38, 40]. The second key step of the mechanism is achieved by the coordination of alkene to the regenerating Pd(II) catalyst, including the water molecule attacking the more substituted olefinic carbon, forming a hydroxyalkyl‐palladium intermediate. In lectures, it was found that the intermediate Sc‐‐‐H−Pd(II)/Sc(III) is formed, and it may act with excess of Sc(III) to reduce Pd(II), and then dioxygen is inserted to make hydroxy‐palladium, which facilitates the Pd catalyst reoxidizing and the methyl ketone generating (Figures 5 and 6). Otherwise, this intermediate may be reduced to inactive palladium(0). Subsequent dioxygen insertion forms the HOO–Pd(II)/Sc(III) intermediate, which may release the HOO− anion and promote the oxidation of the olefinic group [37, 38, 39, 40].

The NMR spectra exhibited sharp peaks in the characterization of the catalytic system of Pd(OAc)_2_/Al(OTf)_3_; these peaks change sharply to appear as resonance between 10 and −10 ppm, their absorbance is clear, and it is indicated that an interaction between Pd(II) and Al(III) cations exists. The result of NMR spectra is supported with DFT calculations, which illustrated the acetate ligands in the Pd(II)/Al(III) system immediately assigned in three forms: (a) heterobimetallic Pd(II)/Al(III) dimer with one acetate bridge, (b) heterobimetallic Pd(II)/Al(III) dimer with two acetate bridges, and (c) original Pd3(OA c)_6_ (Figure 5c) [38].

The Pd(II)/Sc(III) is dimer‐catalyzed Wacker‐type oxidation, which is more efficient than Cu^2+^; the products of the oxidation reaction have been obtained with excellent results. The Pd‐catalyst with trivalent Lewis acid is a good catalytic strategy and could serve as a significant key active species in C—H activation and provide an applicable bioactive molecule in pharmaceutical chemistry. Non‐redox metal ions are used as additives to improve the stability of redox catalysts in homogeneous and heterogeneous catalysis; they also occur in certain active sites of redox enzymes, like Ca^2+^ in the oxygen evolution center of photosystem. Several methods have been published on the catalytic non‐redox carbon dioxide fixation in cyclic carbonate. The new complex of Pd(II) linkage to Sc(III) cation through the di‐acetate bridge makes Pd(II) more electron‐deficient. The improvement of the activity of metal‐like oxo‐intermediates and stoichiometric by catalytic oxidation, which has been investigeted as a new pharmaceutical products could be a great strategy in new drug discovery and development [38, 45, 46, 47, 48].

## 4 The Relationship of the Physic–Chemical Properties of Pd (II) Catalyst With Its Implication

Palladium acts and serves as an excellent catalyst in olefin oxidation, which is a key part in medicinal product developments, e.g., in the oxidation of hydrocarbons containing double/triple bonds, whether aliphatic or aromatic compounds. Palladium is able to absorb more than 900 times its volume of hydrogen. It is not tarnished by the atmosphere at normal temperatures. The absorption of Pd(II) causes both the electrical part and its station to form a metallic or alloy‐like hydride that is formed from hydrogen sources, but Pd‐hydrated could be removed by increased temperature with reduced pressure. When palladium is heated, it combines with several non‐metallic elements. Many compounds in the (M^4+^) state, the (M^2+^) oxidation state, and the (M0) state are also known. Palladium compounds can be prepared in a variety of oxide states [14, 49].

N‐heteroaromatics of a variety of pharmaceutical compounds with highly effective chemical synthesis of intermediates, such as indoles, quinolones, and imidazoles for cross‐coupling with olefins and aldehydes, which led to excellent yields under suitable reaction conditions, were made possible by sophisticated catalytic methods. Palladium, iridium, ruthenium, and rhodium complexes are examples of redox metal ion catalysts that have been discovered to be crucial in this field [14, 50, 51]. More recently, organometal catalysis has been shown to be catalyst or additive ligand with high efficiency for the oxidative olefination of certain N‐heteroaromatic compounds. The mechanism of the relationship between Lewis acids and the active ox/hydroxo metal in oxidation reactions is proposed in Figure 6. The oxo/hydroxo metal acts as an internal base or a nucleophile to abstract a proton or facilitate recombination, leading to the formation of the new C—O bond, like in oxygenation or, in the case of olefination and isomerization, facilitating a subsequent step like beta‐hydride elimination after migratory insertion of an olefin [14, 50, 52].

**FIGURE 7:**
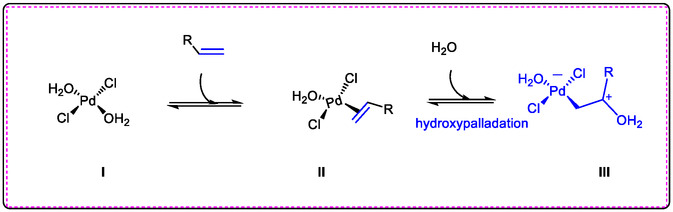
Hydroxy‐palladation in wacker type of oxidation of olefins to aldehydes and ketones actives‐intermediate drugs, its modified form reference [53].

This review provides a relationship between Lewis acids and Pd(II) catalysis reactivity and the recent developments in the olefination of indole derivatives by olefins and the influence of Lewis acids on Pd(II) catalysts. Lewis acid can promote cross‐coupling via regioselective olefination of N‐hetero‐aromatic compounds [13, 48, 49]. Hydroxypalladation has been explored by the mechanism of olefin oxidation reaction with PdCl_2_ in the presence of water; see (Figure 7) [54, 55]. The first step was to coordinate complex **I** with the alkene to form complex **II**. Some of the reporting of common studies recommended that the conclusion step of the reaction is the process of forming complex **III** with the addition of water and palladium‐coordinated olefins, often referred to as palladium hydroxylation of non‐active compounds to produce actual drugs through C—H activation [54, 56, 57, 58, 59].

The investigation of oxidation reactions and the interplay between Lewis acids and active metal intermediates—such as oxo, hydroxo, or hydroperoxy species (M^+^=O, M^+^—OH, M^+^—OOH) and/or hydroperoxide complexes (M^+^—BH—OH)—is a valuable area of study for understanding oxidation reactions in pharmaceutical processing [22, 52].

**FIGURE 8:**
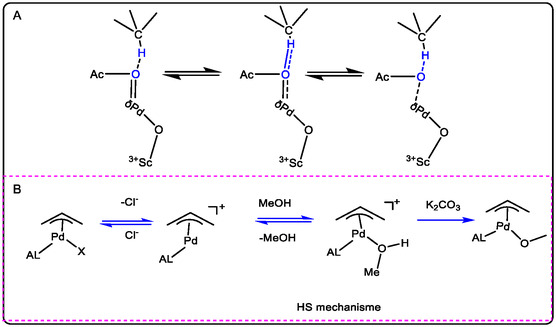
The formation heterobimetallic Pd^2+^ and Sc^3+^ (A) facilitation of the C—H activation and functionalization and insertion dioxygen (B) the Pd‐catalyzed and activation in presence of K_2_CO_3_ in MeOH as the base or using KO*t*Bu in *i*Pr‐OH [52].

The C—H activation systems shown in Figure 8 demonstrate two distinct approaches. In Figure 8A, a Pd/Lewis acid system promotes dioxygen insertion, and the resulting high acidity facilitates alkene functionalization through oxo‐activation to yield a new reactive intermediate. In contrast, Figure 8B shows that when K_2_CO_3_ is used as a base catalyst in methanol, the Pd catalyst enables C—H activation to form an allylic intermediate. To improve upon these methods and accelerate C—H activation via a redox pathway, this study proposes replacing K_2_CO_3_ with Sc^3+^ to form a heterobimetallic Pd^2+^/Sc^3+^ system [22, 60, 61, 62].

## 5 Palladium Catalyzed the Allylic C—H/C—C Oxygenation in Present Lewis Acids

Due to the ubiquity of C—H and C—C bonds in the organic compounds of both production and consumption, the catalytic functionalization of unsaturated hydrocarbons—including long‐chain olefins (alkenes), alkynes, and allylic systems—is of great importance. A key strategy involves the oxidative functionalization of olefinic bonds using molecular oxygen. In previous publications, Pd(OAc)_2_ has been optimized as a catalyst for these transformations. However, it requires a stoichiometric oxidant to regenerate the active Pd(II) species and complete the catalytic cycle. For direct allylic C—H functionalization, suitable terminal oxidants include hypervalent iodine(III) compounds, Cu(II) salts, or quinones, often in combination with aerobic dioxygen to regenerate the Pd(II) catalyst [38, 40, 41, 42].

**FIGURE 9:**
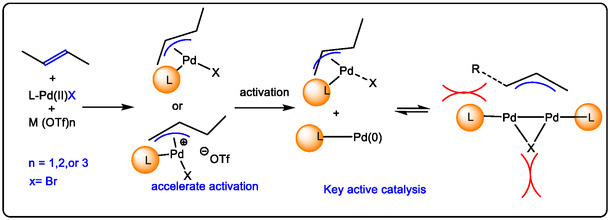
The mechanism of allyl palladium complexes and its activity with pyrazolyl phosphine ligands [63].

This review focuses on recent developments in the oxidation of terminal and internal olefins via C—H and C—C bond activation. Additionally, it covers the oxidative alkylation and arylation of carbon nucleophiles with unsaturated hydrocarbons, their asymmetric variants, and other miscellaneous related reactions. The Pd(II)/Lewis acid (LA)‐catalyzed activation of the allylic C—H bond can proceed via two primary mechanistic pathways: (1) when using Pd(II) salts with alkenes, palladium first coordinates to the non‐polarized *π*‐bond of the alkene (Figure 9). This initiates C—H activation, followed by nucleopalladation. Subsequent β‐hydride elimination, facilitated by an added oxidant, results in dehydropalladation, releasing the product and generating an H–Pd–X species. A new palladium species then coordinates to another olefin molecule to continue the catalytic cycle. (2) The second pathway begins with direct allylic C—H activation. In allylation reactions employing a Lewis acid containing a –OTf moiety, the Pd(II) is reduced to Pd(0). The weakly coordinating nature (low acidity) of the triflate anion promotes the decoordination of Pd(0), a step commonly associated with Wacker‐type oxidation mechanisms. These mechanisms will be explained in greater detail in the following sections [22, 28].

Protonation by a Brønsted acid, followed by the formation of positively charged active metal intermediates, enhances reactivity by facilitating electron transfer. It has been demonstrated that non‐redox metal ions—acting as Lewis acids, in contrast to Brønsted acids—promote highly efficient catalytic oxidations of unsaturated hydrocarbons when paired with a triflate (OTf) moiety (Figure 9) [64]. Particularly, in the oxidation of olefins, the addition of Al(III) as a Lewis acid promotes the generation of a Pd(II) species when a bpym ligand is present. This ligand bridges the Pd(II) center and the Al^3+^ ion via two remote nitrogen atoms. This coordination increases the net positive charge on the Pd(II)‐bpym‐Al^3+^ intermediate and enhances the redox potential of the Pd(II) species. These findings suggest that the Lewis acidity and redox potential of Cu(II) may also play a significant role in versatile Pd(II)‐catalyzed C—H bond activation reactions. Cu(II) is commonly employed as a co‐catalyst to re‐oxidize reduced Pd(0) back to Pd(II). However, studies show that Lewis acids such as Sc(OTf)_3_ and Al(OTf)_3_ exhibit higher reactivity than Cu(OTf)_2_ in these systems. This represents an important modern development in catalysis, highlighting how redox‐active metal ions can improve both oxidation reactions and C—H bond activation in organic synthesis. Furthermore, the effect of solvent in palladium‐catalyzed olefin oxidation has been investigated and appears to produce similar influences across different redox metal ion co‐catalysts [63, 64, 65].

## 6 Pd(II)/Lewis Acid Catalyzed Oxidative C—C Cross‐Coupling Formation via C—H Activation

**SCHEME 1:**
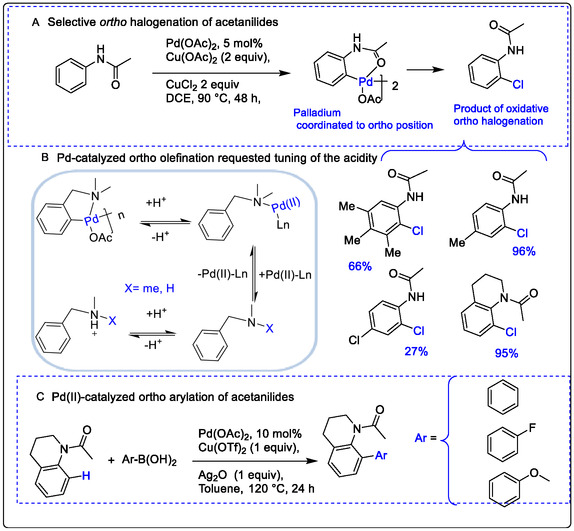
Producing pharmaceutical intermediates by Pd‐catalyzed oxidative C—C cross‐coupling formation. (a) Pd(II) selective ortho halogenation. (b) Pd(II)‐catalyzed and selective ortho olefination (c) Pd(II)‐catalyzed ortho arylation. adpted from references [66, 67] License ID: 1702202–1 is provided by the ROYAL SOCIETY OF CHEMISTRY. Chemical Society Reviews, and Copyright Clearance Center.

The formation of Pd(II) or Pd(0) species is a key step in oxidative functionalization via C—H activation, providing access to versatile catalytic methodologies. In particular, the Heck‐type alkylation pathway offers a suitable mechanism for C—H activation and palladium intermediate formation in Pd‐catalyzed cross‐dehydrogenative coupling. For example, Pd(OAc)_2_‐catalyzed ortho‐halogenation of acetanilides in the presence of CuX_2_ was observed to yield a new mixture of amide products. This represents an interesting approach to C—H activation and halogenation using Pd catalysis. Notably, the ortho‐chlorination proceeds via nucleophilic substitution halogenation, which contrasts with the traditional electrophilic halogenation mechanism (Scheme 1) [63, 64, 65, 66, 67]. Previous studies have reported Heck‐type olefination involving palladacycle intermediates formed through ortho C—H metalation, followed by coupling of arenes with olefins or using aryl halides as substrates (Scheme 1A). Additionally, the N, N‐dimethylaminomethyl group has been employed as a directing group due to its reactivity with various functional groups. In systems where strongly donating ligands are generated in situ under oxidative Pd catalysis, highly acidic Lewis acids have been used as co‐catalysts to enhance electrophilicity in ortho arylation reactions involving Grignard reagents (Scheme 1B). This approach leverages the ability of the amine group to coordinate metal ions and act as a directing group. Reactivity is further influenced by the enhanced acidity under the reaction conditions, leading to dimeric catalytic pathways and enabling the successful synthesis of diverse pharmaceutical intermediates [66, 67].

In Scheme 1C, acetanilide reacted with aryl boronic acids under simple conditions to produce useful biaryls. The nature of Lewis acid in this process is essentially unknown at that moment; it's observed in promoted Pd‐catalyzed alkylation of aryl C—H bond activation [68]. The designed C—C coupling formation is initiated from electrophilic C—H activation and palladation of arenes with the release of Pd(0) species. In other words, Pd(II), while terminated by reductive elimination with both aromatic C—H bonds and aryl boronic acid, is a nucleophilic reaction that has been chivied. The Pd‐catalyzed C—C formation by bonding a C—C bond between two nucleophiles would require a stoichiometric oxidant such as Lewis acid Cu(OTf)_2_ and an acidic property in medium. Lewis acids are required to enhance the electrophilic ability of the Pd(II) species [69]. That permits the rate of C—H cleavage and should be faster than the homo‐coupling of arylboronic acid with an ensured catalytic sequence. To properly do this, the condition must be carefully optimized and, using Ag_2_O as an additive (Scheme 1C).

**FIGURE 10:**
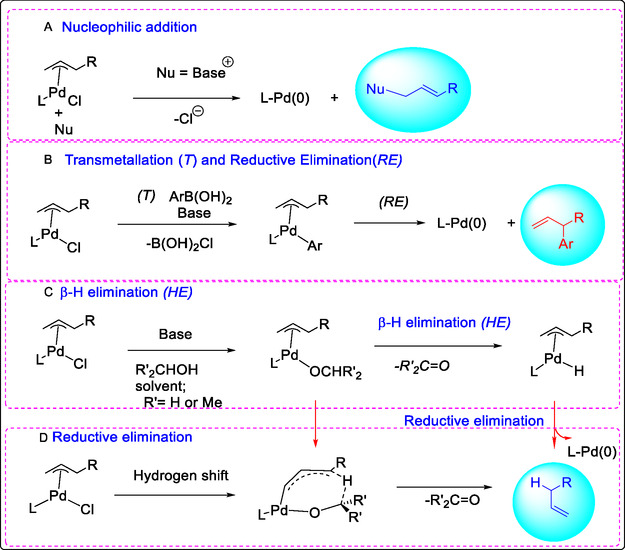
(A) Nucleophilic addition to produced an olefin, (B) reductive elimination with the nucleophilic coupling arylation (Ar) organoborane in the Suzuki−Miyaura reaction. (C) Using alcohol solvents as a good source of hydrogen, which allows H^+^ transfers to supported of obtaining good results. (D) *β*‐H and reductive eliminations, or by hydrogen shift [60, 70, 71, 72].

**FIGURE 11:**
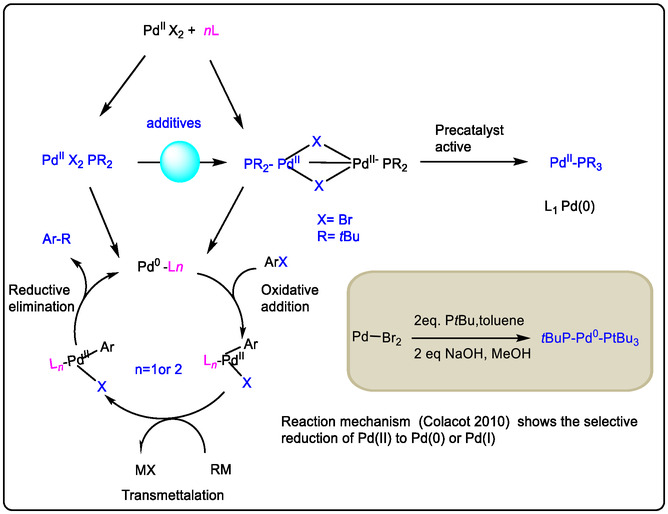
Formation of Pd(0)‐monophosphine by the activation of Pd‐halide‐bridged, modified from reference [52], License Number 6212751438563, is provided by the American Chemical Society, Journal of the American Chemical Society, and Copyright Clearance Center.

The mechanism of conversion of Pd(II) to Pd(0) under catalytic conditions is suggested to involve Lewis acid co‐catalysis, occurring in alcoholic solvents with aerobic oxygen. These conditions might also facilitate oxidation to produce the (Pd–OH–base) complex intermediate. Whatever, there are several different studies of reduction/oxidation pathways, which have been proposed on the basis of the olefin conversion and could be good intermediates for the development of pharmaceutical incorporation and medicinal delivery in Figure 10. Despite the fundamental importance of Pd(II) reduction to catalytically active Pd(0) in catalytic cycles, the mechanistic details of this decomposition pathway remain inadequately understood. Proposed mechanisms suggest that reduction is mediated by base, alkoxides, alcohol, or organometallic reagents—particularly cross‐coupling partners such as organozinc, organomagnesium, or boronates—as illustrated in Figure 11. Recent investigations into the role of acidity in Pd‐catalyst efficiency have provided novel insights and directly contributed to the development of improved precatalyst designs [68, 69, 70]. These studies support a stepwise mechanistic framework, consistent with established literature: (a) Nucleophlic attack by a base (Nu) on an η^3^‐allyl palladium complex, generating an olefin of the type CH_2_‐(Nu)‐CH‐CHR see Figure 10A,B). (b) Transmetalation with an organoborane nucleophile (Ar), followed by reductive elimination in the Suzuki–Miyaura reaction. (c) In α‐arylation reactions, alcoholic solvents can serve as a hydrogen source, facilitating olefination and leading to improved yields. (d) The final step may proceed via a stepwise β‐hydride elimination and reductive elimination, or through a concerted hydrogen shift [60, 70]. To gain further insight into the stereospecificity and reaction mechanism proposed in the literature, control experiments were performed under the optimized conditions. Interestingly, these conditions yielded all four possible diastereoisomers in good yields. Using the oxidation of anethole as a model reaction, the interconversion of 4‐methoxy allylbenzene was studied. This olefin oxidation was investigated using organometallic catalysts [71, 72].

## 7 Pd(0)‐Monophosphates Generated by Activation Pd(II)‐Lewis Acid Catalyst

The active metal of a Lewis acid binds to Lewis basic heteroatoms in dimeric catalyst systems. This concept has been realized, for example, by employing a Lewis acid with a palladium catalyst to assist in the reduction of Pd(II) intermediates to Pd(0) in oxidation reactions [37]. The formation of Pd(0) is a key elementary step for understanding palladium catalysis in olefin oxidation, cross‐coupling, C—H activation, and all catalytic processes related to Wacker chemistry. Notably, in the presence of phosphine ligands such as PtBu_3_, the reduction from Pd(II) generates the oxidation state Pd(I), rather than Pd(0). Experimentally, the reduction of Pd(II) to Pd(0) has been investigated using Lewis acidic metal reagents such as Cu(II) [72]. Furthermore, precatalysts such as {Pd(μ‐Br)(PtBu_3_)}_2_ and (tBu_3_P)_2_Pd have shown active catalytic oxidation that depends on the reductive conversion of Pd(II) to Pd(I) by stoichiometric reducing agents. Additionally, in existing Pd(II) catalyst systems, phosphine‐containing species are critically important. For instance, the mechanism involves the formation of a Pd(I) bromide dimer, alongside a Pd(II)Br_3_ dimer side‐product; these species have been characterized and identified (see Figure 11).

The catalytic potential of palladium complexes has been extensively studied in key cross‐coupling reactions, including α‐arylation, Buchwald–Hartwig amination, and Suzuki–Miyaura coupling [73]. The synthesis of a new Pd(I) bromide dimer precatalyst (Figure 11) has also been achieved, delivering enhanced catalytic activity [74, 75, 76]. In recent years, the development of novel Pd−PtBu_3_ precatalyst systems has attracted significant attention as a challenging advance for cross‐coupling reactions, following its initial identification in 1998 [77]. The dimeric Pd(I) complex {Pd(μ‐Br)(PtBu_3_)}_2_ is classified as an L_1_Pd(0)‐type precatalyst and exhibits exceptionally high catalytic activity in a variety of modern cross‐coupling reactions [52, 78, 79].

Figure 11 shows method created by the Colacot group of the synthetic procedures for the formation of Pd(I) bromide dimer {Pd(μ‐Br)(PtBu_3_)} [61, 64, 76, 80]. Pd(II) catalyst monomers and their Pd(I) μ‐allyl dimer were organized with the Suzuki−Miyaura reaction. Damian P. Hruszkewycz and his co‐authors explored the relationship between the monomeric and the dimeric forms of palladium compounds by using both experiment and theory. The formation of dimers activates Pd(IPr)(*η* ^3^‐allyl)Cl and IPr−Pd(0) by disproportionation of the (μ‐allyl)(μ‐Cl)Pd_2_(IPr)_2_‐type. However, Pd(I) μ‐allyl dimer is formed, and the IPr−Pd(0) active species is removed from the reaction mixture. This identification was a novel method by the addition of NaOH in MeOH for treating Pd(cod)(Br)_2_ (cod = 1,5‐cyclooctadiene) with 1 equiv of PtBu_3_ [79, 80].

In some literature, the catalytic effect of Nolan‐type precatalysts, (η^3^‐allyl)Pd(PR_3_)Cl, and Colacot or Shaughnessy‐type precatalysts, (η^3^‐allyl)Pd(NHC)Cl, has been reported and demonstrated. Their catalytic reactivity is influenced by several key factors: (a) the rate of formation of the catalytically active L‐Pd(0) species.

(b) The competition between protonation of the L‐Pd(0) species and its reaction with the starting materials. This protonation can lead to the formation of a Pd(I) μ‐allyl dimer, which also serves as an efficient pathway for removing L‐Pd(0) from the reaction mixture. Colacot and others have used these insights to develop improved precatalysts. In systems of the type (η^3^‐allyl)Pd(L)Cl (where L = NHC or PR_3_), the formation of active Pd(0) and/or Pd(I) dimer species was shown to be effective. However, the (μ‐allyl)(μ‐Cl)Pd_2_(L)_2_ dimer itself remains under‐explored, and its kinetic role in catalytic cycles is still not fully understood [52].

## 8 Lewis Acids Promoted Oxidative Dehydrogenation of N‐Heterocyclic Compounds

The activity of a Pd(OAc)_2_ catalyst for the oxidative dehydrogenation of saturated C—C bonds in N‐heterocycles was enhanced by support with non‐redox metal ions. Catalyst characterization via UV–vis and NMR spectroscopy, along with recent studies, has confirmed that the key active species is a heterobimetallic dimer complex, consisting of Pd(II) and the Lewis acid Al(OTf)_3_ in acetonitrile [37, 38, 40, 81]. The highly catalytic effects are mediated with strong Lewis acids like Sc^3+^ and/or Al^3+^ can interact with N‐heterocyclic compounds, which was impressively observed in dehydrogenation, oxidation and isomerization. Adding 2 mM of Lewis acid such as Al (OTf)_3_ was an adequate choice of acidity that warded the dehydrogenation reaction and achieve a high yield (Table 1). The strong acidity of non‐redox metal ions was compared with amines, N‐heterocyclic, cyclic ketones are mostly neutral and have much more robust C—H bonds. For example, the selection of some substrates such as 4‐t‐butyl‐cyclohexanone for oxidative dehydrogenation, and the results are summarized in (Table 1). Clearly, that the promotional effect of Pd^2+^/Zn^2+^ is lower influence and provides a low yield 14.1% of 4‐t‐butyl‐cyclohexenone without t‐butyl‐phenol formation, however, it is still slightly but better than using Pd(OAc)_2_ alone [83, 84, 85, 86, 87].

**TABLE 1:** The oxidative dehydrogenation of N‐heterocyclic compounds via C—C bond activation by Pd(II) catalyst.^a^

Entry	Substrate	T/h	Conv.%	Products	Yield %
1	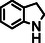	6	77.4(34.4)	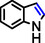	71.0(32.6)
2	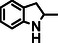	3	97.6(51.0)	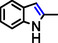	74.0(43.2)
3	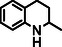	24	62.1(46.9)	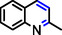	50.9(37.6)
4	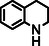	12	59.8(26.2)	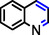	55.1(23.6)
5	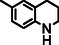	24	47.0(23.3)	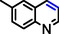	34.0(18.7)

a **Condition of reactions**: Solvent, acetonitrile 5 mL, and Zn(OTf)_2_ (2 mol%, 2 mM) added to Pd(OAc)_2_ (1 mol%, 1 mM), 100 mM of substance, O_2_ balloon, 80°C–90°C, in entry 4 substrate (50 mM) and entry 3 Substrate (20 mM). The conversion and yield calculation by using GC analysis and the data in parenthesis result of control the experiments and test Pd(II) alone without Lewis acid ion [82].

In organic chemistry, Pd‐catalyzed C—C coupling reactions are among the most important catalytic methods. They have been widely employed for the oxidation of various unsaturated hydrocarbons, particularly olefins. Kinetically, the oxidative formation of C—C bonds via the activation of C(sp^2^)—H to C(sp^3^)—H bonds is disfavored in reactions catalyzed by redox metal ions. The performance of Pd(II)‐catalyzed oxidation and conversion via C—H activation can be accelerated by the addition of strong Lewis acidic metals, such as Al(III). This enhancement allows for the efficient generation of oxo‐ and hydro‐intermediates. For example, ketones were obtained with a 28.7% yield. However, this product was a mixture, primarily consisting of 4‐*tert*‐butylcyclohexenone, with tert‐butyl phenol also formed in a 10.3% yield [88]. Table 1 illustrated that the significance effect of Lewis acid, e.g., Sc(III), provided promotional good effects; it's found in the increasing of the yield and selective oxidative dehydrogenation of N‐heterocyclic [36].

The magic work of Lewis acids with Pd(II) catalyst in the chemical conversion of cycloaliphatic compounds to their aromatic conjunctives. The oxidative dehydrogenation that converted the cyclo‐ketones to phenols was developed following the abstraction of hydrogen atoms; the improvement was due to adding trivalent Lewis acid under aerobic conditions (Table 2).

**TABLE 2:** The oxidative dehydrogenation of cyclic ketone by Lewis acid/Pd(OAc)_2_ catalyst.

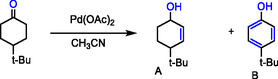				
Entry	Lewis acid	Conv %	Yield A%	Yield B%
1	—	8.9	4.6	—
2	Zn(Otf)_2_	14.1	7.6	—
3	Al(Otf)_3_	68.2	28.7	10.3
4	Sc(Otf)_3_	64.3	37.7	15.7

**Conditions**: Lewis acid 16 mM added to Pd(II) 8 mM, and mixture in CH_3_CN 3 mL, and then 4‐*t‐*butyl‐yclohexanone 80 mM was added, 20 atm O_2_, 80°C, 24 h [ 36, 82].

Similarly, using the Pd(II) as a redox metal catalyst and desired in the oxidation dehydrogenation of the indoline with trivalent Lewis acid, for improving the efficiency of the Pd(II) catalyst by adding an access ratio of Sc(III) to the mixture reaction of the 4‐t‐butyl‐cyclohexenone, this ratio is spatially with samples selected of cyclo‐ketones (Table 2). However, using the access ratio of Sc(III) (Lewis acid) to Pd(II) acetate in another chemical operation is useless; for example in the oxidation and/or isomerization of olefins, the best ratio (2:1) of dimer Sc(III)/Pd(II) catalyst was investigated [38, 83, 85, 89, 90].

## 9 Lewis Acids Promoted Wacker‐Type Oxidations

In this study, we also compiled methods and figures related to the oxidation of olefin derivatives, with a focus on Wacker‐type processes. These include oxidative cross‐coupling olefination, dehydrogenation, oxygenation, oxidative amidation, and arylation. A common strategy employs redox metals, such as Pd(II), in conjunction with a Lewis acid. This is exemplified in the oxidation of olefins and plant oils, where the transformation is achieved through one or more of the following key steps: a) C—H bond activation, where hydrogen loses its valence electrons, altering the oxidation state of the carbon center. b) Direct oxygen insertion into the C—H bond. c) Hydrogen atom abstraction (H^+^), facilitating the combination of the carbon radical with oxygen.

### 9.1 Oxyfunctionalization of Internal Olefins of Vegetable Oils

**FIGURE 12:**
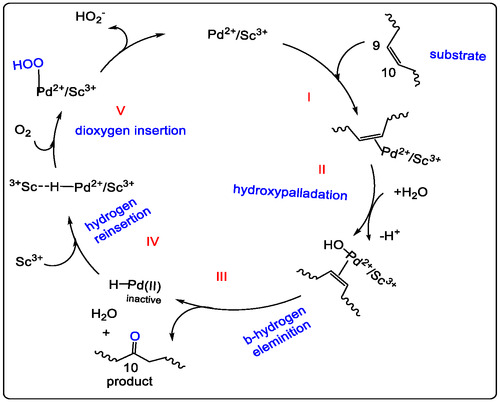
The proposed Wacker type oxidation mechanism and Sc(III) promote Pd(II) catalyzed oxyfunctionalization of vegetable oils and others unsaturated fatty acids, adapted from references [40], License Number 6212680269824, is provided by the American Chemical Society, Journal of Agricultural and Food Chemistry, and Copyright Clearance Center.

In this work, we provide some important Pd‐catalytic methods with triflate‐metal, Sc^3+^ and/ or Al^3+^ selected for supported Pd(II)‐catalyzed C—H bond activation, with Lewis acids serving as non‐redox metal ions, which plays a significant role in oxyfunctinalization of vegetable oils. Lewis acids such as Sc^3+^ improved the reactivity of Pd(II)‐catalyzed oxidation of vegetable oils via C—H bond activation (Figure 12) [73].

It is a good opportunity for catalyst design in organic synthesis for producing natural pharmaceutical products. The proposed mechanism of Walker‐type oxidation showed that the formation of Sc(III)…H–Pd(II) intermediate is developed, which disallows reduction elimination of the Pd(II)‐hydride with Lewis acid ligands. Hydroxypalladation is carried out with β‐Proton elimination by adding the inhibitors of the inactive palladium black, and that facilitates the insertion of the dioxygen by palladium hydride, which supports Wacker‐type oxidation of internal olefins in vegetable oils (see Figure 12) [38, 39, 40, 66, 82].

The efficiency of palladium‐catalyzed oxyfunctionalization of unsaturated fatty acids to their corresponding ketone derivatives was significantly enhanced by the addition of non‐redox metal ions, particularly Sc(III). Using a Pd(II)/Sc(III) catalyst system, substrates including methyl oleate, methyl palmitoleate, and methyl linoleate underwent Wacker‐type oxidation. This yielded the corresponding ketone‐fatty acid methyl esters with > 99% conversion and excellent yields (up to 92.5%). The procedure efficiently oxidized alkadienes and other unsaturated fats. Using a catalyst system of Pd(OAc)_2_ and Sc(OTf)_3_, these substrates were converted via a Wacker‐type oxidation mechanism to their conjugated intermediate products. This work demonstrates a broadly applicable and highly efficient method for the oxyfunctionalization of various fatty acid substrates, as detailed in Table 3 [40].

**TABLE 3:** Wacker‐type oxidation of different vegetable oils by Pd(OAc)_2_ with the Sc(OTf)_3_ in acetonitrile /water (v/v, 2.7/0.3).

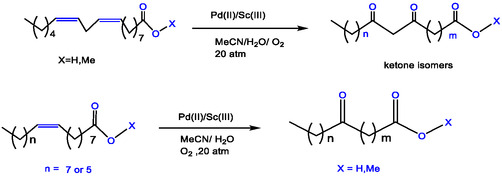				
Entry	Substrate	Time	Main product isolated	Yield %
1	Methyl oleate	18 h	Methyl 10‐oxooctadecanoate	92.5
2	Oleic acid	18 h	10‐oxooctadecanoic acid	75.8
3	Methyl(Z)‐hexadec‐9‐enoate	18 h	Methyl10‐oxohexadecanoate	91.2
	Methyl palmitoleate			
4	Methyl linoleate	24 h	Methyl9,11‐dioxooctadecanoate	83.4
5	Linoleic acid	24 h	9,11‐dioxooctadecanoic acid	67.3

**Conditions**: Mixture of acetonitrile 2.7 mL/ H_2_O 0.3 mL, Sc(III) 4 mM added to Pd(II) 2 mM, 100 mM of substance, 80°C, O_2_ 20 atm. The yield is determined by ^1^HNMR in CDCl_3_ except entry 3 in CD_3_CN [40, 41, 87].

The transformation of methyl linoleate via oxidation, isomerization, and hydroxylation into conjugated derivatives was investigated using a simple Pd(OAc)_2_ catalyst combined with a Lewis acid dimer. This review introduces the catalytic oxidation of vegetable oils—specifically methyl linoleate and its derivatives—into corresponding keto fatty acids/esters, employing a Pd(OAc)_2_/Sc(OTf)_3_ catalyst system, which holds broad industrial relevance. It was demonstrated that incorporating Lewis acids such as Sc(III) into a simple Pd(II) catalyst significantly enhances the oxidation activity of vegetable oils as renewable biomass sources in a MeCN/H_2_O solvent. Preliminary mechanistic studies, along with earlier work, confirm that the in situ‐generated heterobimetallic Pd(II)/Sc(III) dimer acts as the key species in vegetable oil oxidation, proceeding via a [1, 3] hydrogen shift mechanism that involves a formal Pd(II)/Pd(IV) catalytic cycle [91, 92, 93, 94].

**FIGURE 13:**
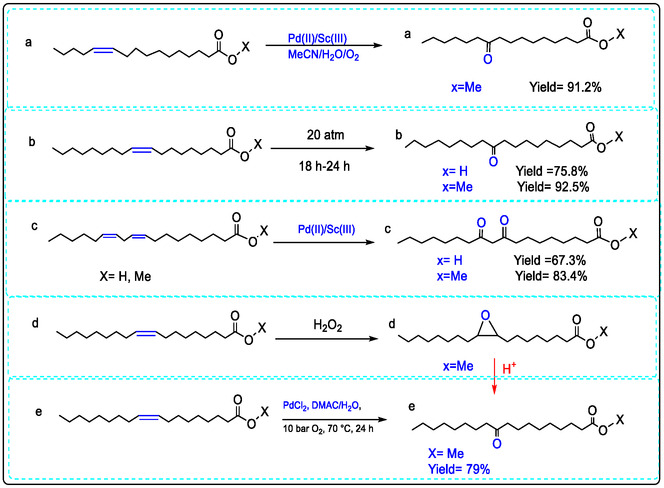
The oxidation of biomass oil/fatty acids by Pd(II)/Lewis acids catalyst (a) Pd/LA‐catalyzed oxidation of a mono‐ (double bond) of methyl oleate/Oleic acid or double (double bond) like in methyl linoleate/linoleic acid and compared the result with (b, d, and e) reactions, which they reacted under different conditions involving high pressure [9, 40, 103].

This work has presented as a modification of Wacker‐type oxidation for the transformation of unsaturated fatty acids/esters to the corresponding keto‐fatty acids/esters, in which Cu(II) cation was replaced by common Sc(OTf)_3_ as a common nonredox metal ions, which utilized a novel Pd(II)/Lewis acid (LA)‐catalyzed fatty acids, and in this case the ratio of 2:1 is investigated [10, 95, 96, 97, 98, 99, 100, 101, 102]. Follow‐up products of fatty acid oxidation have daily importance, for example, as plasticizers [98]. In Figure 13a,b, methyl ester was oxidized to methyl 9(10)‐ketostearate (91.2% yield) by a Wacker oxidation by Pd/Lewis acid and oxygen as the re‐oxidant, and similarly, oxidation of methyl linoleate to 9,11‐diketonstearate fatty acids (83.4% yield) (Figure 13c). The oxidation of the oleate methyl ester to the keto‐methyl ester, using PdCl_2_ in dimethylacetamide (DMAC) under an O_2_ atmosphere, proceeded with a 79% yield (Figure 13e). Plant oils are considered a source of unsaturated compounds, and their reactions using efficient Lewis acid catalysts have been reviewed. Chemo‐enzymatic epoxidation with H_2_O_2_ is another example, as shown in (Figure 13d) [9, 40].

### 9.2 PdCl_2_/Cu^2+^‐Catalyzed Oxidative Acetalization of Terminal Alkenes

**FIGURE 14:**
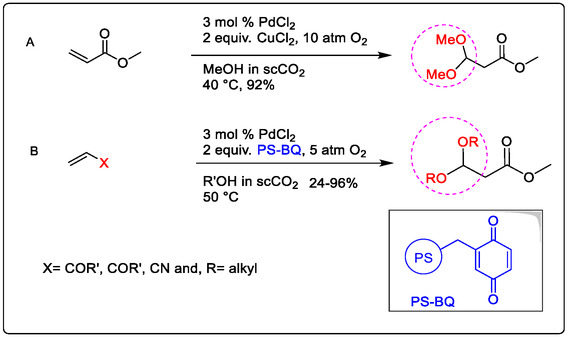
Pd(II)‐catalyzed acetalization of terminal olefins with alcohols using different co‐catalyst in ScCO_2_; (A) CuCl_2_, (B) polystyrene‐supported benzoquinone (PS‐BQ) [104].

Developing homogenous catalysis for organic synthesis, Pd/Lewis acid as a dimer catalyst has been a long‐term objective of our group. We have explored some oxidation reactions of alkynes by Pd catalyst in (Figure 14) [38]. This demonstrates the improved catalytic efficiency of Wacker oxidation using a Lewis acid, while also focusing on advanced and efficient aerobic oxidation methods. Notably, the use of Cu^2+^ as a co‐oxidant promotes Pd‐catalyzed oxidation via C—H activation of double bonds. The Wacker‐type oxidation is a crucial reaction for olefin functionalization, specifically the acetalization of olefins with alcohols. This is important because acetals like *β*‐keto acetals and *β*‐cyanoacetates are key intermediates in the synthesis of fine chemicals for pharmaceuticals and industry. In 1999, Zhao, Yang, and co‐authors reported the Pd‐catalyzed acetalization of terminal olefins using aerobic oxygen in the presence of either CuCl_2_ or polystyrene‐supported benzoquinone (PS‐BQ) (Figure 14) [104, 105].

**SCHEME 2:**
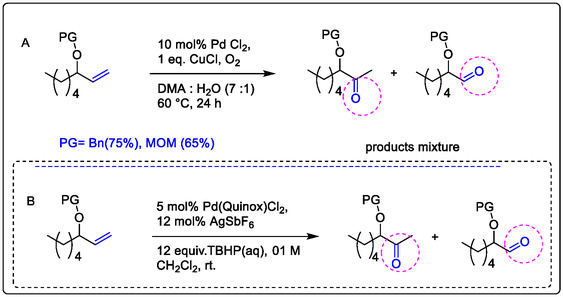
(a) Pd(II)/CuCl and O_2_ for obtaining methyl ketones and aldehydes, (b) Pd^2+^/TB HP‐mediated Wacker‐type oxidations of allylic alcohols as a good pharmaceutical intermediates [106, 107].

The formation of Pd(1) and/or Pd(0) is the key step of catalysis in the oxidation reaction, which is C—H bond activation with many versatile catalytic reactions. Particularly, Heck‐type pathway alkylation provides a suitable mechanism for the C—H activation and palladium intermediate formation. In a certain way, some reports illustrated the Wacker‐type oxidations of allylic alcohols by Tsuji‐type conditions for the desired methyl ketone that have been investigated. King and co‐workers obtained methyl ketone and aldehyde from allelic alcohols by using protected allylic alcohols as substrates (Scheme 2) [106, 107].

### 9.3 Palladium‐Catalyzed Oxidation of Terminal Olefins With AIBN and O_2_

**FIGURE 15:**
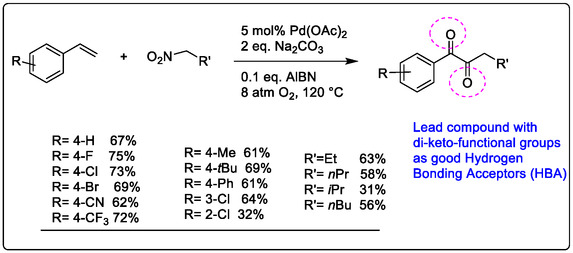
Pd‐catalyzed carbonation di‐ketonization of terminal alkenes with nitro alkanes in drug discovery [103].

In this section, one of the most challenging targets of modern organic synthesis is the homogeneous catalytic methods, like Pd‐catalyzed carbonation‐diketonization of alkene. Three types of chemical processes achieved the pathway: (1) functionalization of the CN bond, (2) C—C bond formation reactions via CN bond activation, and (3) cleavage and release of sulfonamides, amides, or aniline as the coupling partners of diketonization [108]. However, the development of the carbonation diketonization reaction of terminal alkenes was improved by adding 2,2‐azobisisobutyronitrile (AIBN), which facilitates the methyl radical generated from nitromethane in the presence of Pd(OAc)_2_ and Na_2_CO_3_, all of which are suggested for improving drug discovery processes; see the details in Figure 15.

### 9.4 Pd(OAc)_2_/Zn(OTf)_2_ Catalyzed Oxidation‐Cyclization of Alkynes

**FIGURE 16:**
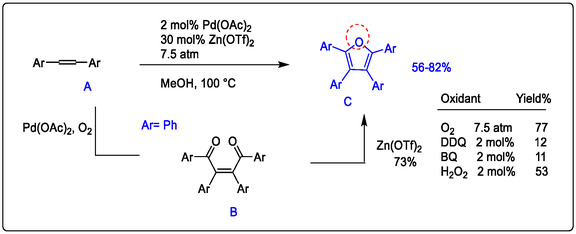
Pd(OAc)_2_/Zn(OTf)_2_‐catalyzed oxidation‐ cyclization of alkynes as pharmaceutical intermediates, modified from references [109].

This review is concerned with the oxidation and cyclization of alkynes as simply unsaturated hydrocarbons. Specifically, the synthesis of furan derivatives examined various Lewis acids toward this type of oxidation, including using Zn(OTf)_2_, which was shown to be highly effective with a palladium catalyst. While the other oxidants, such as DDQ, were very sluggish (see Figure 16) [109].

**FIGURE 17:**
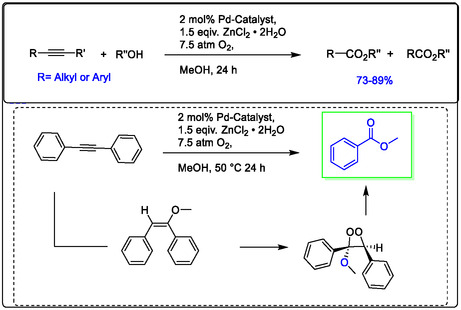
Pd‐catalyzed cleavage internal triple bonds in alkynes to produce the pharmaceutical intermediates with aerobic conditions [104].

The oxidative cleavage of the internal triple bonds in alkynes was investigated by Pd^2+^/Zn^2+^ with molecular O_2_. In Figure 17, illustrated that the influence ratio is distinctly increased by adding Lewis acid ZnCl_2_·2H_2_O to samples of Pd(OAc)_2_, significantly increasing the catalytic efficiency of palladium and cleavage of the internal triple bonds, the possible pathway cleavage reaction with molecular O_2_ illustrated the influence adding Lewis acid ZnCl_2_·2H_2_O to samples of Pd(OAc)_2_ significantly increasing the catalytic efficiency of palladium and cleavage of the internal triple bonds, the possible pathway cleavage reaction with molecular O_2_ [108, 110].

### 9.5 Pd‐Catalyzed Oxidation of the Internal Olefins With Molecular O_2_

**FIGURE 18:**
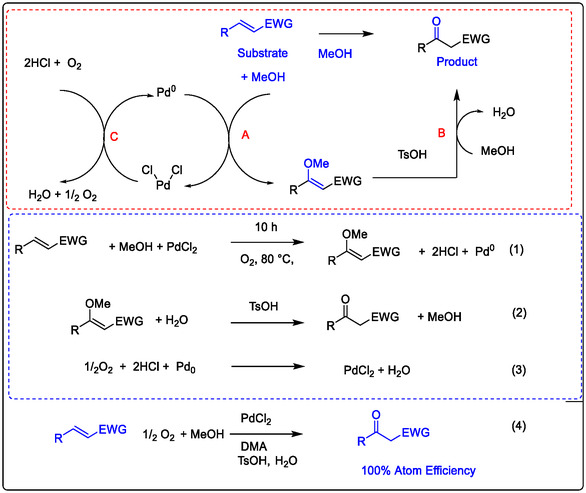
Pd‐catalyzed oxidation of the internal olefins to ketones with molecular O_2_; Mechanism proposed pathway catalyst system in improving of pharmaceutical intermediates [111].

In our previous work, we have developed a highly efficient catalytic system consisting of Pd(OAc)_2_ with Lewis acid [39], despite PdCl_2_·2H_2_O with DMA, TsOH, and MeOH showing good applicability for the aerobic oxidation and conversion of the internal olefins to ketones. The oxidations of various electron‐deficient olefins are selected in our review, which illustrates the internal electron‐deficient olefin oxidized to the corresponding ketones, such as β‐keto products, in overly high yield with 99% selectivity. Here we demonstrate the selective oxidation of electron‐deficient olefins in the presence of O_2_ as a green oxidant, thus leading to the efficient production of a wide range of ketones from olefins (Figure 18) [111].

### 9.6 Pd‐Catalyzed Oxidative C—H Olefination

**FIGURE 19:**
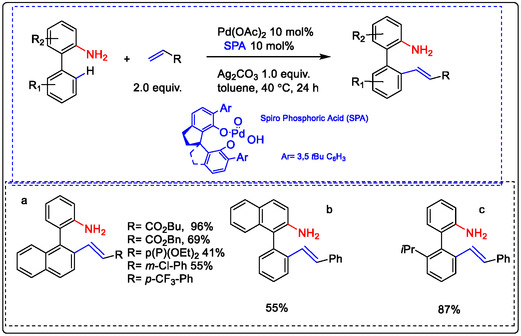
Pd(II)‐catalyzed free‐amine‐directed ortho‐selective olefination of pharmaceutical intermediates via C—H activation and determination of geometrical relationships between the substrates and yields, like in b and c. modified from reference [112], License ID: 1702192‐1, is provided by the Royal Society of Chemistry.

The modified chiral spiro‐phosphoric acid (SPA) ligand, in combination with Pd catalysis, enabled selective meta‐substitution directed by a free amine group. This modification significantly enhanced the reactivity of the palladium catalyst. Consequently, a Pd(II)‐catalyzed C—H olefination reaction efficiently produced chiral biaryl‐2‐amines in excellent yield and with high enantioselectivity. This methodology was applied in drug discovery and development, yielding several target products (a–c in Figure 19) [113]. While the formation of five‐, six‐, and seven‐membered rings is typically governed by thermodynamic stability, in this case, the reaction was directed by an amino group to favor kinetically controlled metallacycle transition states, leading to ortho‐functionalization. The observed enantioselectivity was further enhanced by the narrow geometry of the SPAs. As illustrated below, this constrained environment promoted a specific interaction with the substrate intermediate. Moreover, the stabilized acetate ion of palladium is based on the cyclometallation deprotonation/oxidative type C—H activation. Thus, the determination of geometrical relationships between the substrate, catalyst, and functional group and C—H bond covering in the other substrates as pharmaceutical intermediates [112].

**FIGURE 20:**
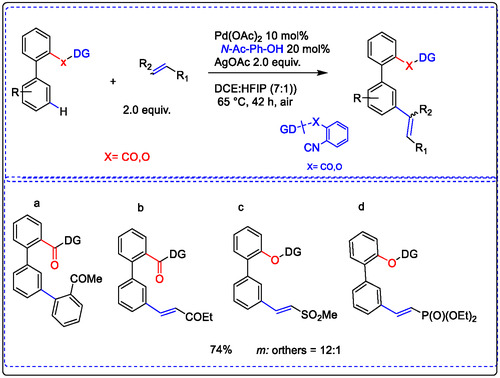
Pd‐catalyzed olefination of biphenyl carboxylic acids and phenols via meta‐substation [112, 114].

In 2016 Wajid Ali and co‐authors explored the distal C—H olefination of phenols and biphenyl carboxylic acids by the Pd(OAc)_2_ catalyst. Delivering highly selective meta‐olefination products from the substrate attached with a carbonyl and/or nitrile group as the weak coordination linker group. The reaction processed well with the Pd catalyst in the presence of MPAA ligand oxidant, N‐Ac‐Ph‐OH, and/or AgOAc to enhance meta‐olefination products (a–d in Figure 20) [112].

Both substrates showed almost similar reactivity under the optimized conditions, which validated the principle of the selectivity–reactivity paradigm. For meta‐selective olefination, the Lewis ligands completely control the reaction involvement of the directing group. At the same time, a computational study suggested that the acetate bridge form of a Pd–Ag bimetallic as an active complex involved, linked with the 2‐pyridone molecule, lowers the energy barrier. C—H activation olefination through a cyclometallation–deprotonation pathway [114].

## 10 Lewis Acid Promoted the Oxidative Coupling Olefination With Pd(OAc)_2_ Catalyst

Transition metals in complexes contain a moiety (Otf), have been employed as Lewis acid in redox reactions, which promoted stoichiometric oxidation reactions with Pd(II) catalyst in different procedures of organic synthesis and offering high catalytic activity for oxidation, and C—H bond activation [115, 116, 117]. The Brønsted acid or base has been employed in heterogeneous redox catalysts to improve their stability and reactivity, in similar impacts to those in homogeneous, Lewis acid‐accelerated Pd(II) catalyzed olefins and vegetable oils and oxidative coupling reactions, which were also investigated (see Table 4) [120, 121, 122, 123].

**TABLE 4:** The oxidative olefination of indole with methyl acrylate by Pd(OAc)_2_.

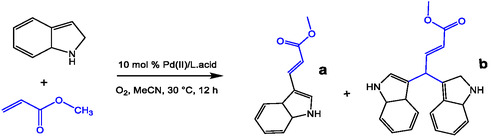			
Entry	Pd(II)/Sc(III) mol %	solvent	Yield(a)% and (b)%
1	10/‐	MeCN	6 (a)
2	10/20	MeCN	63 (b)
3	5/10	MeCN	45 (b)
4	10/10	MeCN	34 (b)
5	10/30	MeCN	55 (b)
6	10/20	MeCN	7 (b)

**Conditions**: in CH_3_CN (2 mL), Lewis acid (20 mol%) was added to Pd(OAc)_2_ (10 mol%), O_2_ balloon, 30°C for 12 h. Yield of compound(a) and (b) were isolated and determined by NMR [118, 119].

Naturally, certain non‐redox metal ions are known to be involved in the oxidative processes in the system of photosynthesis, where Ca(II) is one component of the active Mn_4_CaO_5_ catalyst. Using Pd(OAc)_2_ with Ca(OTf)_2_ as a catalyst system accelerates the oxidative coupling of indoles with olefins through C—H and dioxygen activation, but it is not better than using Sc(OTf)_3_ [118, 119, 124]. Since 2014, studies have explored the efficacy of incorporating non‐redox ions with transition metals in dimeric catalysts. This led to a focused investigation into Lewis acid/Pd catalyst systems. For instance, adding a strong Lewis acid like scandium(III) triflate to a redox metal such as Pd^2+^ or Fe^2+^ in a mixture has been shown to successfully enhance C—C and C—H activation, as well as oxidation reactions, and has been employed in numerous synthetic processes. The synergistic relationship between transition metals, particularly noble metals like Pd^2+^, and Lewis acids in heterobimetallic catalysts has been investigated. DFT calculations confirm that adding Sc(OTf)_3_ to Pd(OAc)_2_ stabilizes a new heterobimetallic Pd(II)/Sc(III) intermediate, influenced by the conformational energies of the OAc^‐^ and OTf^‐^ anions. This active Pd^2+^–OAc^‐^–OTf^‐^–Sc^3+^ bridge species, characterized by NMR and UV–vis spectroscopy, is identified as the key catalytic site in modified Wacker‐type oxidations. Previous studies demonstrate that Lewis acids significantly improve the reactivity of Pd(II)‐catalyzed oxidative coupling reactions, particularly in C—H bond activation. For example, in the olefination of indoles, the proton transfer step is facilitated by a catalyst system combining Sc(OTf)_3_ and Pd(OAc)_2_. DFT calculations used to determine the configuration energies of these intermediates reveal a decrease in the overall activation energy for the oxidative cross‐coupling reaction [32, 38, 118].

The oxidation region selectivity has been observed by using non‐redox metals with palladium catalysis in different catalytic applications. Similarly, synthesis of the moiety of Co (II) as redox metal ion with Lewis acid cation and the result formed is phenoxide ligand, which has great effects in oxidation. The water with cobalt‐metal catalyst plays significant role in oxidative synthesis, due to the additives of Lewis acid and localization of the redox at the metal center, which can also improve the reactivity of ion‐Pd‐catalyst. However, the redox metal ion like MnII in the oxidation reaction is not active like oxo‐metal cations bound to Mn=O and/or O*μ‐*oxo ligands, which could be used in synthetic cobalt oxide, which could be useful for catalysis in oxidation reactions caused by the incorporation of the bridge of acetate groups with Lewis acid. A new approach may replace the Pd‐catalyst to control the site‐selectivity of C—H activation, and that was reported by Yu and co‐workers [119].

**FIGURE 21:**
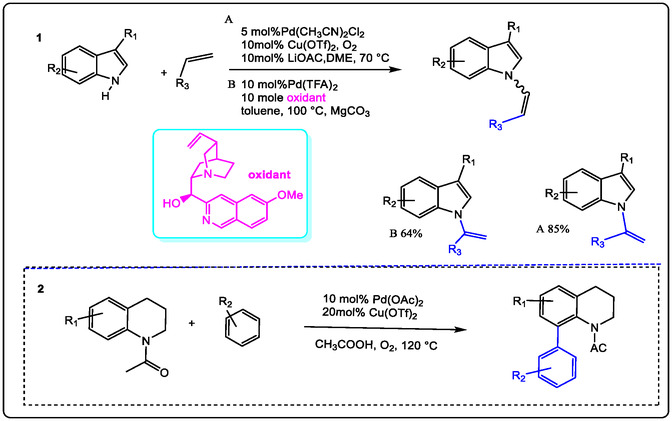
Regioselective‐cross coupling reactions by Lewis acid (1) with quinine as oxidants ligand, (2) Lewis acid with Pd(AOc)_2_ catalyzed cross coupling of N‐acetyl tetrahydroquinolines with arenes [125].

The other achievements with palladium chemistry in C—C‐coupling reactions include palladium‐catalyzed oxidative C—C alkenylation of indoles with olefins, as demonstrated by Wei‐ping Su, who realized the N‐alkenylation of indoles with a variety of different alkenes, allylic ethers, and, in some cases, giving the double bond isomerization. Cardinally to the literature and the modified procedure, using the Ba(OTf)_2_, Na(OTf), K(OTf), Ca(OTf)_2_, and Sr(OTf)_2_ with redox‐catalyst. Furthermore, in condensation synthesis, the ethylenediamine was investigated by using the appropriate metal triflate salt and purified the product by recrystallization. Figure 21 also illustrates the aerobic oxidation of tertiary amines for C—N/C—C coupling, as reported by Wang in 2012. In this work, oxidative olefination of THIQs was achieved with high enantioselectivity using a redox catalyst mixture containing Cu(OTf)_2_ and quinine as a chiral organocatalyst (1). The process also featured a regioselective cross‐coupling reaction (2), mediated by a palladium catalyst and a non‐redox copper species acting as a Lewis acid [125].

From Figure 21, it is proposed that the indole N‐heterocycle coordinates to the Pd^2+^ cation, enabling olefination via nucleophilic addition of the C2=C3 bond to acrylates—a significant reaction for producing pharmaceutical intermediates.

**FIGURE 22:**
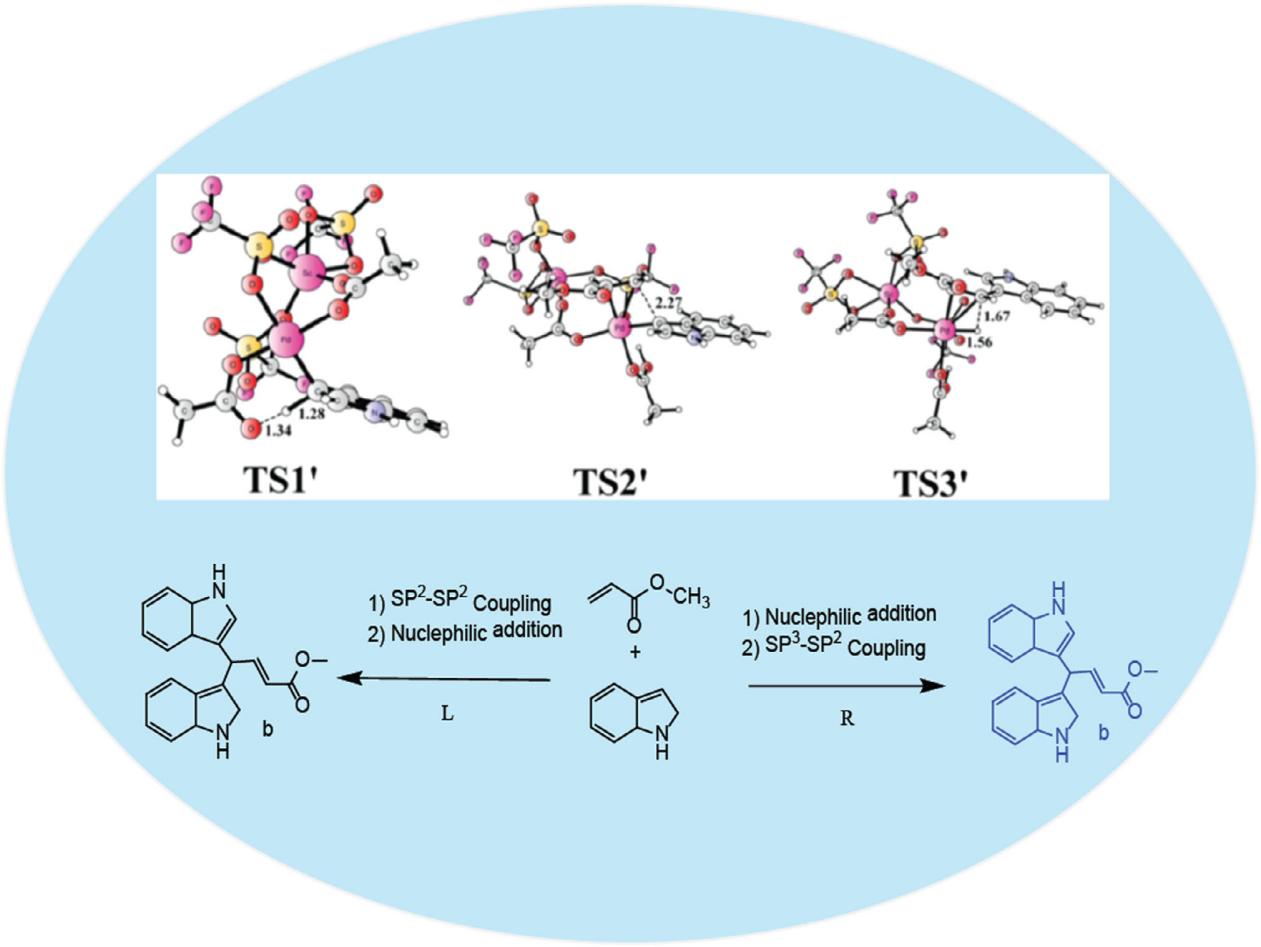
Possible C—C‐coupling pathway and formation of bis(indolyl) propanoic esters by nucleophilic addition [116, 118], its adpted from reference [118], License ID: 1701563‐3, is provided by the Royal Society of Chemistry, Organic & Biomolecular Chemistry.

Density functional theory (DFT) calculations provide further details of oxidation/isomerization that enhance the results. The calculations support a proposed mechanism involving a heterobimetallic [Pd(II)/Sc(III)] complex. In this dimeric structure, two triflate (OTf^‐^) anions bridge the Pd(II) acetate units. The mechanism was investigated computationally. It proceeds through a concerted metalation‐deprotonation (CMD) step at the indole's C3 position, generating intermediate Int1 via transition state TS1’ (Figure 22). This C3‐arylation metalation step is crucial, as the calculations show the bimetallic system catalyzes the formation of the major product. Prior theoretical studies in the literature are in strong agreement with experimental results for this C3‐arylation pathway. Subsequent steps involve: 1) coordination and carbopalladation of a second methyl acrylate molecule (see TS2’, Figure 22), 2) a C—H activation step, and finally 3) *β*‐hydride elimination (see TS3’, Figure 22). The resulting Pd(II)–hydride and other Pd‐intermediate complexes were characterized by NMR and UV–vis spectroscopy [126, 127, 128, 129]. Several years ago, the use of various organometallic catalysts for olefin oxidation emerged as an important development. Among these, palladium has gained considerable attention due to its good selectivity in C—H activation. For example, Pd catalysts have demonstrated significant effects in olefin oxidation, leading to extensive and advanced applications, particularly in achieving meta‐selective functionalization of electron‐rich aromatic rings. Nevertheless, this area remains one of the most difficult tasks and a major challenge in this active field of research [61, 130, 131, 132]. Using palladium catalysts in organic chemistry is not new; Pd(II) complexes have found widespread applications, but the support of Lewis acids in olefination oxidation is a qualitative development in the pharmaceutical field. Although a number of reports have been published, with no limited to applications in cross coupling reactions, and it's not fully recognized, several conjugation reactions have emerged in chemistry and won the Nobel Prize by using the Pd(n) catalyst in chemistry: (a) Suzuki coupling reaction and its application of halide with boronic acid/ester in the presence of a palladium (0) catalyst (which is an important class of coupling reactions and the production of caparratriene, which was used for the treatment of leukemia) [133, 134, 135, 136]. (b) The Wurtz or Wurtz–Fittig reaction (Pinacol coupling reaction) [137, 138, 139, 140, 141], (c) the Glaser–Eglinton reaction, two terminal alkynes are coupled by a copper (II) salt [142]. (d) In Sonogashira coupling reaction using the Pd/Cu redox catalyst of coupling reaction between terminal alkynes with halides, and similarly, the alkyne group goes through the halogenation in alkynes trimerization, both starting materials for polymers that are easy to access with the common intermediate such as alkynes [82, 125, 126, 127, 128, 129, 130]. (f) The garter work investigated by Mizoroki–Heck on arylation reactions, has used aryl‐halides and olefins in the presence of organometallic catalysis as Lewis acids [94, 143, 144, 145, 146].

**SCHEME 3:**
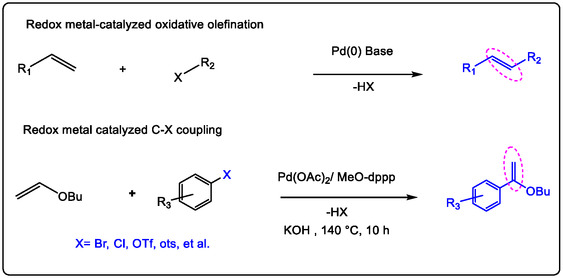
The Pd(II)/ base catalyzed coupling arylation–Heck reaction [94, 143, 144, 145, 146].

In the present work, the Lewis acid/redox metal ions are considered to be more convenient for medical research in the near future to set C—H and/or C—C‐activation, which is suggested for the synthesis of the new applicable chemicals for assaying bioactivities and behaviors of the inhibitor growing as candidate drugs. The olefination of alkyl halide with olefins is investigated, which offered the initial steps of the reaction by using a palladium catalyst and forming an active catalyst, Pd(0)‐base, as the intermediate of key C–halide bond activation. It proceeds following the role of the “base‐assisted oxidative addition” mechanism (see Scheme 3) [147, 148, 149].

**SCHEME 4:**
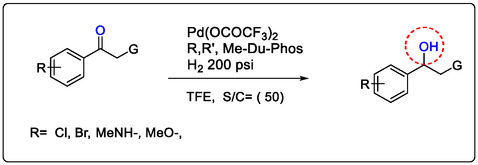
Asymmetric redox‐hydroxylation of ketones to alcohols [147, 148, 149].

Homogeneous Pd‐catalysts are supported by LA for fine chemical synthesis due to their important advantages: (i) Ready formation of palladium complexes with an extensive diversity of organic ligands, including triflates, bivalates, and monovlates Lewis acids; (ii) Palladium complexes such as Pd(OAc)_2_ are green catalysts and found with different precursors; (iii) Pd‐catalyzed reactions are easy to run in ordinary equipment. (iv) Palladium catalyst in the functional group tolerance of synthetics and transformations is very useful and truly impressive. The homogeneous Pd(II)‐catalyzed hydrogenation of imines and hydroxylation of olefins compounds introduced, (see Scheme 4) [150, 151, 152, 153].

**FIGURE 23:**
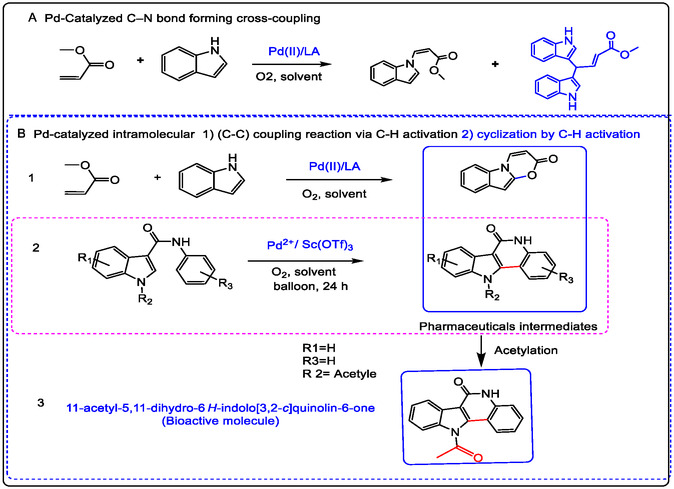
Pd(II) /LA catalyzed (a) C‐N bond forming and olefination (b) Pd(II)‐catalyzed C‐C/C‐H activation cyclization like in12 and 3, which the coupling/cyclization oxidative reaction of indoles with acrylates for producing N‐hetero‐pharmaceutical intermediates like 11‐acetyl‐5,11‐dihydro‐6H‐indolo[3,2‐c]quinolin‐6‐one [154].

In 2005, Zhou and co‐workers reported the palladium(II)‐catalyzed hydrogenation of ketones to produce phenols and alcohols. This homogeneous catalytic system was highly enantioselective and played a significant role in producing pharmaceutical intermediates and aiding drug discovery research. The reaction was particularly effective in the presence of weakly coordinating anions, such as M–OTf and CF_3_CO_2_^‐^, which led to complete conversion of the reactants [150]. Previous studies have extensively explored the applications of palladium in various organic syntheses and transformations, often involving the addition of nucleophiles via optimized methods. For example, Pd(OAc)_2_ has been used for the oxidative coupling of indole with olefins (Figure 23A,B). The efficiency of palladium continues to be leveraged in the synthesis of pharmaceutical products, especially in the presence of Lewis acids. Furthermore, palladium's relatively economical price and low toxicity make it a suitable candidate for redox metal‐catalyzed coupling reactions in pharmaceutical synthesis. Experiments have shown that the synergistic combination of palladium and a Lewis acid is a successful strategy, advancing both academic research and technological applications in drug development. Historical literature also illustrates that palladium has long been widely used as a catalyst in oxidation and coupling reactions. Consequently, palladium is frequently recommended for developing catalytic strategies within the chemical and pharmaceutical industries [118].

The acetylation modification of the intermediate compound in Figure 22 for producing a bioactive molecule (11‐acetyl‐5,11‐dihydro‐6H‐indolo[3,2‐c]quinolin‐6‐one), belonging to the indoloquinoline family of fuzed heterocyclic systems, which has a significant interest in medicinal chemistry due to their potent biological activities, shows a variety of functional groups ordering for binding sites with biological targets. Adding an acetyl group to this molecule would be supported by its affecting properties like solubility, absorption, distribution, metabolism, and excretion (ADME). Palladium, in combination with a Lewis acid catalyst, enables C—H activation under aerobic conditions using dioxygen. This catalytic system has been employed for the synthesis of polycyclic heteroarene skeletons (Figure 23B) [155]. Very recently, Pd/Lewis acid employed for oxidative cyclization via C—H/C—C bond activation has been significantly challenging for developments in pharmaceuticals now. That is optimized under low pressure of molecular dioxygen to hypothesize polycyclic heteroarene skeletons, and the result is investigated with good advantage of atom economy in organic synthesis under simple conditions [154].

## 11 Anti‐Markovnikov Oxidation and Transformations of Terminal Alkenes to Aldehydes as a Pharmaceutical Intermediates by Pd(II)‐Catalyst

**SCHEME 5:**
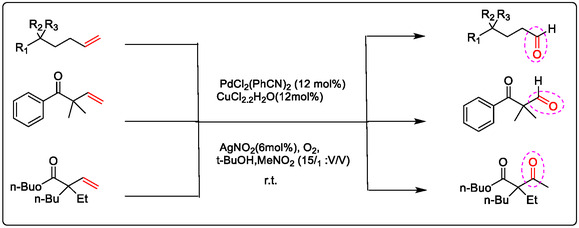
Pd‐catalytic transformations and oxidation terminal alkenes following anti‐Markovnikov and aldehyde‐selective Wacker‐type oxidation [11].

In 2016, Kim and co‐authors reported the catalytic oxidation of alkenes to aldehydes as potential drug candidates using an AgNO_2_/PdCl_2_(PhCN)_2_/CuCl_2_·2H_2_O catalyst system. The reaction was investigated with optimal overall yield and selectivity. The presence of AgNO_2_ as a nitrite source significantly increased catalytic efficiency, while its exclusion severely impeded oxidation. This result strongly supported the critical role of the nitrite source. However, we endeavored to clarify a deficiency in the understanding of the Lewis acid property of the nitrate species and its positive effect on the reaction outcome (see Scheme 5). Furthermore, the study highlighted the synthetic impact of aldehyde‐selective Tsuji−Wacker oxidation on the reactivity and functionalization of alkenes. The process involved selective functionalization at the allylic or homoallylic position. It was also illustrated how the resulting aldehydes, following the anti‐Markovnikov rule, serve as valuable intermediates for producing fine chemicals in drug discovery [11].

The literature on Pd(OAc)_2_/ligand‐assisted (LA) homogeneous systems catalyzing the hydrocarboxylation and methoxycarbonylation of phenylacetylene has been investigated.

**SCHEME 6:**
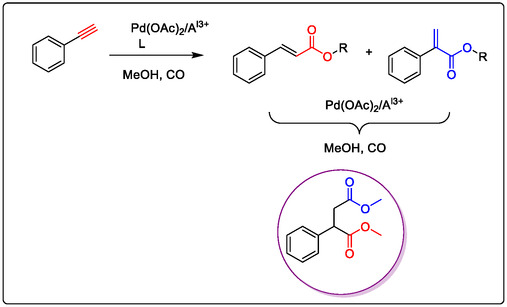
Diester functional groups formation by methoxycarbonylation as bioactive intermediates [154].

This study demonstrates a Pd‐based catalyst system that employs Al(OTf)_3_ as a co‐catalyst and PPh_3_ as a supporting ligand for the methoxycarbonylation of alkenes. The product obtained is either the methyl ester or its corresponding carboxylic acid, depending on the reaction medium additives. Carbon monoxide is used to direct the extension of the carbon chain (Scheme 6) [156, 157].

## 12 Conclusion

This study explores methods for the Pd(II)/Lewis acid‐catalyzed Wacker‐type oxidation of olefins, alkynes, natural oils, and the C—H/C—C activation‐based alkylation of N‐heteroaromatics. The oxidative olefination and alkylation reactions used to synthesize drug intermediates demonstrate that adding non‐redox Lewis acids to Pd(OAc)_2_ results in higher‐quality oxo‐chemical products with improved catalytic efficiency. The Pd(II)/Lewis acid system exhibits superior oxidation potential and efficiency compared to noble metals, even in the presence or absence of oxidant ligands like Cu^2+^, making it valuable for producing pharmaceutical intermediates.

Furthermore, this work summarizes recent research on palladium‐catalyzed functionalization of fine industrial and pharmaceutical molecules, including derivatives such as bis(indolyl)methanes. The strategic modification of oxidized molecules presents a powerful avenue to enhance their binding affinity at target sites and optimize their Structure‐Activity Relationship (SAR). This includes critically important physicochemical and pharmacokinetic properties such as solubility, absorption, distribution, metabolism, and excretion (ADME). Building on this principle, this study demonstrates the utility of the PdII/LA catalytic system for the multi‐step synthesis of an active pharmaceutical ingredient (API) precursor. The synthesized compounds are strategically designed with modifiable sites, providing a versatile chemical handle to refine them into viable drug candidates [157].

Therefore, we propose that future research should focus on this integrated strategy: applying the efficient, green procedures of the PdII/LA system to produce novel scaffolds, followed by targeted chemical modification. These dual approaches will advance the lead compounds and identify the optimized candidates with supported enhanced bioactivity and drug properties [40].

## Author Contributions

A.M.S. contributed to writing – the original draft conceptualization, methodology, investigation, validation, and data curation. S.A. contributed to writing – review and editing, supervision, funding acquisition, and project administration. S.K.A. contributed to writing – review and editing, supervision, and funding acquisition. [Correction added on 4 April 2026 after first online publication: The funding information has been updated.]

## Funding

This study was supported by Türkiye Bilimsel ve Teknolojik Araştırma Kurumu (1059B212200167); Deanship of Scientific Research, King Khalid University (R.G.P.2/47).

## Conflicts of Interest

The authors declare no conflicts of interest.
